# Structure-Based Design and In Silico Evaluation of a Lipophilic Cyclooctanoyl- Derivative as a Renin Inhibitor: Lessons from Withdrawn Aliskiren

**DOI:** 10.3390/ijms262311398

**Published:** 2025-11-25

**Authors:** Dimitrios Pavlos, Errikos Petsas, Filippos Panteleimon Chatzipieris, Thomas Mavromoustakos, Christos T. Chasapis

**Affiliations:** Laboratory of Organic Chemistry, Department of Chemistry, National and Kapodistrian University of Athens, Panepistimiopolis Zografou, 15771 Athens, Greece; errpets@chem.uoa.gr (E.P.); fchatzip@chem.uoa.gr (F.P.C.); tmavrom@chem.uoa.gr (T.M.)

**Keywords:** renin, molecular docking, molecular dynamics, N-CDAH, ADME, toxicity prediction, withdrawn drug, safety profile, antihypertensive drug design, computational protein design

## Abstract

Renin, a key aspartic protease central to the renin–angiotensin–aldosterone system (RAAS), remains a therapeutic target for hypertension despite the withdrawal of the only approved direct renin inhibitor, Aliskiren, due to unfavorable drug–drug interactions and safety concerns. Here, we report a computational protein design-driven evaluation of (S)-3-((3-(1H-imidazol-1-yl)propyl)amino)-2-(((S)-1-carboxy-2-(cyclooctanecarboxamido)ethyl)amino)-3-oxopropanoic acid (N-CDAH), a novel lipophilic cyclooctanoyl- derivative, as a next-generation renin inhibitor scaffold. This scaffold was designed based on the rationale of leveraging the carnosine like backbone while optimizing lipophilicity and metabolic stability. Pharmacokinetic, ADME, and toxicity predictions (SwissADME, pkCSM) revealed greater predicted aqueous solubility, enhanced metabolic stability, and significantly reduced off-target liabilities compared with Aliskiren (specifically, non-inhibition of major CYP isoforms). Molecular docking (AutoDock Vina binding affinity: −8.08 kcal/mol; Maestro Induced Fit Docking score: −11.149 kcal/mol) and molecular dynamics simulations confirmed favorable binding interactions, conformational adaptability, and complex stability within the renin active site. To contextualize its performance within the broader chemical space, the diastereomeric analog of N-CDAH as well as structurally related compounds identified through SwissSimilarity were also examined using computational workflow. The MD analysis (200 ns) demonstrated that the inhibitor is anchored via a dual stabilization mechanism: hydrophobic enclosure coupled with persistent ionic interactions. These integrative in silico results highlight the potential of this derivative to overcome Aliskiren’s pharmacological shortcomings, providing a strong computational rationale for experimental validation and underscoring the role of structure-based drug design in antihypertensive drug discovery.

## 1. Introduction

Hypertension imposes a growing global health challenge, affecting over 1.3 billion individuals and driving substantial cardiovascular morbidity and mortality [1,2]. The renin–angiotensin–aldosterone system (RAAS) lies in the heart of blood pressure homeostasis, with renin catalyzing the conversion of angiotensinogen to angiotensin I, a rate-limiting step that ultimately produces vasoconstrictive angiotensin II [3,4].

Direct renin inhibition emerged as a promising strategy, leading to the development and approval of Aliskiren. Despite its potent suppression of plasma renin activity, clinical application uncovered critical limitations. The pivotal ALTITUDE trial revealed that, in high-risk patients with type 2 diabetes already receiving angiotensin-converting enzyme inhibitors or angiotensin receptor blockers, Aliskiren failed to improve cardiovascular or renal outcomes. Instead, it was associated with a higher incidence of adverse events, including hyperkalemia, hypotension, renal dysfunction, and an unexpected increase in nonfatal strokes, prompting early termination of the study [5]. These safety concerns, along with Aliskiren’s low oral bioavailability, extensive CYP3A4-mediated metabolism, and suboptimal tissue distribution, ultimately led to its market withdrawal [6,7,8].

Computational strategies can be particularly valuable for optimizing such drug based leads, as they allow rapid exploration of structure–activity relationships, prediction of drug-likeness, and identification of molecular determinants of binding affinity [9].

**Figure 1:**
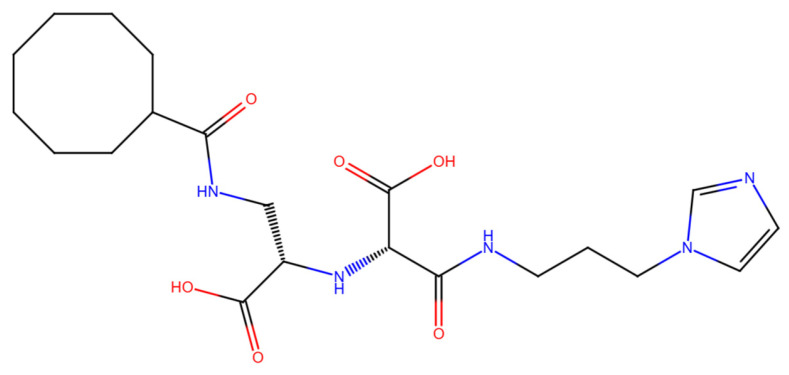
Chemical structure of N-CDAH, drawn in ChemDraw 12.0.2 (blue: nitrogen atoms and their hydrogens, red: oxygen atoms and their hydrogens). The lipophilic cyclooctanoyl- group is designed to enhance membrane permeability, while the polar backbone and the imidazole ring preserve key hydrogen bonding and ionic interaction capabilities. Functional groups—including carboxylates-, amide bonds, hydroxyls-, and imidazole- nitrogens—are highlighted to indicate multiple potential contact points with the renin active site.

To leverage and enhance these properties, we engaged in - drug-based molecular engineering and designed a lipophilic cyclooctanoyl- derivative; (S)-3-((3-(1H-imidazol-1-yl)propyl)amino)-2-(((S)-1-carboxy-2-(cyclooctanecarboxamido)ethyl)amino)-3-oxopropanoic acid (N-CDAH) (Figure 1) [10]. Here, we present the computational characterization of this novel tripeptide derivative as a direct human renin inhibitor. Using a combination of molecular docking, binding free-energy evaluation, molecular dynamics simulations, and in silico ADME/Tox profiling, we compare its molecular docking affinity, binding interactions, dynamic stability, and ADME Tox profile against Aliskiren’s reported data to assess its potential to overcome previous shortcomings and inform the development of safer antihypertensive agents [11]. In addition to the parent compound, the diastereomeric analog of N-CDAH and a set of structurally related compounds identified through the SwissSimilarity platform were also examined to contextualize its performance within the broader chemical space.

This integrative computational workflow not only provides mechanistic insights into ligand–renin interactions but also illustrates the potential of druge-based molecular engineering in the design of safer and more effective antihypertensive agents, aligning with current advances in computational protein design.

## 2. Results

### 2.1. Pharmacokinetic and Drug-likeness Properties; Comparison with Withdrawn Aliskiren

All comparative values for Aliskiren refer to its documented post withdrawal profiles, as sourced from regulatory summaries and peer-reviewed literature. This ensured that the benchmarking framework reflected clinically relevant pharmacological limitations of Aliskiren while situating the designed cyclooctanoyl- derivative within a computational drug design context.

Computational ADME predictions for N-CDAH, based on SwissADME and pkCSM platforms, indicate a drug like profile dominated by high polarity and hydrophilicity. According to SwissADME, the molecule has a molecular weight of approximately 451.52 Da, a high topological polar surface area (TPSA ≈ 162.65 Å^2^), and 14 rotatable bonds. LogP is estimated at −0.44, indicating pronounced hydrophilicity. This polarity is reinforced by multiple hydrogen-bonding groups (5 donors and 8 acceptors), which underlie its aqueous solubility (log S_ESOL = −1.10; classified as “very soluble”), but limit passive membrane permeability. As a result, the cyclooctanoyl- derivative is predicted to have low gastrointestinal (GI) absorption, no blood–brain barrier (BBB) permeability, and P-glycoprotein (P-gp) substrate behavior. Importantly, the compound is predicted not to inhibit any major cytochrome P450 isoforms (CYP1A2, 2C19, 2C9, 2D6, 3A4), suggesting a significantly reduced risk of drug–drug interactions. With respect to drug-likeness, it satisfies Lipinski’s rule of five with a single violation (number of N or O atoms > 10), while Veber’s and Egan’s rules are violated due to the high TPSA and number of rotatable bonds. Consequently, its predicted oral bioavailability score is low (0.11) [12].

The design of N-CDAH was guided by known structural requirements for high-affinity renin inhibition. The cyclooctanoyl- moiety was selected to mimic and strengthen the deep hydrophobic interactions of Aliskiren’s aliphatic region, improving pocket encapsulation and residence time. Thus, N-CDAH represents a rational evolution of the Aliskiren scaffold, maintaining essential recognition features while optimizing charge distribution, hydrogen-bonding geometry, and hydrophobic fit.

To address potential conformational heterogeneity, it is important to note that N-CDAH contains a substantial degree of torsional flexibility, with 14 rotatable bonds as predicted by SwissADME. Based on established x-angle rotamer libraries for drug fragments, each l-aspartyl derivative residue is expected to adopt approximately three energetically favorable side-chain rotamers, while the terminal histaminyl- like moiety can access two to three preferred orientations. In addition, the N-terminal cyclooctanoyl- group is characterized by multiple low-energy conformations typical of medium-sized carbocycles. Taken together, these considerations suggest that approximately 25–40 energetically accessible side-chain rotameric combinations exist for the scaffold under physiological conditions. The conformation presented in Figure 1 corresponds to the global minimum-energy geometry, obtained following MMFF94 minimization and used as the starting point for subsequent docking procedures. This representation reflects a plausible low-energy state; however, the full conformational space was explored during flexible docking and molecular dynamics, ensuring that alternative rotamers were not neglected in the binding analysis.

Similarly, pkCSM predictions support these findings, reporting a molecular weight of 451.52 Da, a LogP value of approximately 0.36, and a log water solubility (log S) of –2.892, which also categorizes the cyclooctanoyl- derivative as highly soluble. The predicted intestinal absorption is low (18.7%), and the molecule is not expected to cross the BBB (logBB = −1.276). Like SwissADME, pkCSM also predicts that the molecule does not inhibit CYP enzymes. Total clearance is predicted to be moderate (log 0.58). Collectively, both tools converge on a profile of a very polar, water-soluble compound with limited oral absorption, driven by high TPSA, conformational flexibility, and efflux recognition [13].

N-CDAH is therefore characterized by a unique balance of lipophilic (cyclooctanoyl-) and hydrophilic domains. In contrast, Aliskiren is heavier, less polar, and more lipophilic (C_30_H_53_N_3_O_6_; MW ≈ 551.8 Da). SwissADME predictions for Aliskiren indicate 4 H-bond donors, 7 acceptors, TPSA ≈ 146.1 Å^2^, and LogP ≈ +4.15 Thus, while both compounds share poor oral bioavailability and P-gp recognition, the designed derivative benefits from substantially higher solubility and reduced CYP-mediated liabilities, reflecting a strategy to improve systemic safety [14].

#### Key Comparative Parameters

Compared with Aliskiren (MW ≈ 551.8 Da, LogP ≈ +4.15, TPSA ≈ 146.1 Å^2^), the designed cyclooctanoyl- derivative (MW ≈ 451.5 Da, LogP ≈ −0.44, TPSA ≈ 162.7 Å^2^) is smaller, more polar, and substantially more hydrophilic. The novel compound also exhibits a slightly greater hydrogen bonding capacity (5 donors and 8 acceptors vs. 4 donors and 7 acceptors for Aliskiren), which contributes to its enhanced solubility but limited gastrointestinal permeability. Both molecules share common pharmacokinetic limitations, including low oral bioavailability, absence of blood–brain barrier permeation, and susceptibility to P-glycoprotein efflux. However, unlike Aliskiren, the cyclooctanoyl- derivativeis predicted not to inhibit any major cytochrome P450 isoforms, suggesting a reduced risk of metabolic drug–drug interactions. Despite its lower bioavailability score (0.11 vs. ~0.55 for Aliskiren, with reported clinical absorption of ~2.5%) [15], the improved aqueous solubility and metabolic stability of the cyclooctanoyl- derivative represent a strong design success and may provide an advantage for future optimization and formulation strategies (Table 1).

In summary, both compounds are characterized by high polarity and limited oral bioavailability. However, the designedcyclooctanoyl- derivative offers improved solubility and metabolic stability compared with Aliskiren, representing a computationally guided attempt to balance safety with efficacy while addressing known pharmacological shortcomings.

### 2.2. Molecular Docking Using AutoDock

#### Docking of N-CDAH to Human Renin (AutoDock Analysis)

Docking simulations were conducted with AutoDock 4.2, which employs the Lamarckian genetic algorithm and remains a widely validated platform for protein–ligand interaction studies [16]. The cyclooctanoyl- derivative (N-CDAH) was positioned into the deep catalytic cleft of human renin (PDB ID: 2FS4) with a calculated binding free energy of –8.08 kcal/mol. Consistent with renin’s established structural organization as a bilobal aspartic protease, the catalytic dyad (Asp33 and Asp219) is located between two β-sheet domains, while a flexible flap of loop residues contributes to ligand recognition and stabilization. The active site features a spacious, predominantly hydrophobic cavity, including the S1 and S3 subsites, which form a so-called “superpocket” that is exploited by high-affinity renin inhibitors through hydrophobic or aromatic substituents [6,17,18].

In the present AutoDock model, the imidazole moiety residue is oriented toward the S1/S3 region, nestled between Tyr78 and Phe119. This positioning allows extensive aromatic stacking and hydrophobic contacts, key features observed in known renin–inhibitor complexes.

The most favorable docking pose revealed several stabilizing interactions (summarized in Table 2). π-π stacking was observed between the ligand and aromatic residues Tyr78 (S3 subsite), Phe114, and Phe119 (S1 subsite), which collectively anchor the ligand within the hydrophobic cavity. Hydrophobic van der Waals contacts were further established with Pro42, Val106, Pro113, and Ala117, providing additional stabilization through close packing. Importantly, His56was predicted to be within hydrogen-bonding distance of the ligand’s polar moieties, suggesting a potential polar or π-cation interaction that could contribute to catalytic inhibition [19,20].

These binding features—aromatic stacking with Tyr78/Phe119, hydrophobic occupation of the S1/S3 superpocket, mirror the interaction patterns observed for potent renin inhibitors in structural studies [17,19]. Furthermore, ligand occupation of the hydrophobic S3/S1 region is a recognized determinant of affinity enhancement, as it maximizes lipophilic stabilization while maintaining polar contacts with catalytic residues [6,20].

In summary, AutoDock analysis indicates that N-CDAH adopts a deep-binding conformation within the catalytic cleft of renin, stabilized by complementary hydrophobic and aromatic interactions in the S1/S3 superpocket. This binding pattern supports its potential as an effective renin inhibitor, consistent with established principles of rational renin inhibitor design.

**Figure 2:**
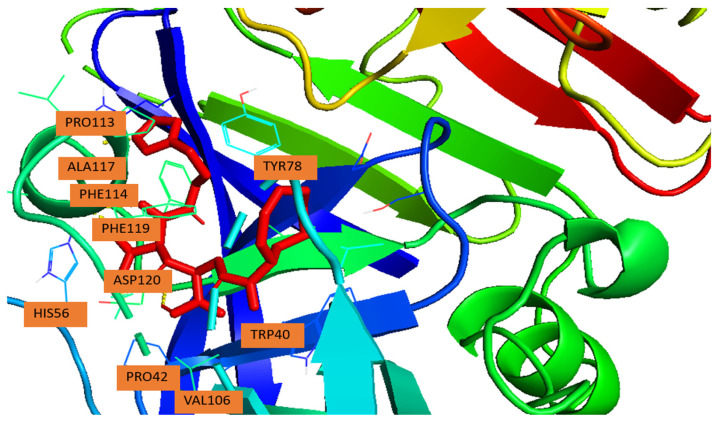
Binding pose of N-CDAH (yellow) within the active site of human renin (PDB ID: 2FS4). The ligand occupies the hydrophobic S3/S1 sub-pocket and forms π-π stacking interactions with aromatic residues Tyr78, Phe114, and Phe119. Additional hydrophobic contacts are observed with. Pro42, Val106, Pro113, and Ala117. The polar residue His56, lies within potential hydrogen bonding distance from the ligand’s polar moieties.

Figure 2 illustrates the binding pose of the ligand in the renin active site, while Table 2 details the amino acid residues and their interaction types.

### 2.3. Molecular Docking of N-CDAH with Renin (PDB: 2FS4). Induced-Fit Docking (IFD) Methodology in Maestro

The Induced-Fit Docking (IFD) protocol in Maestro was applied to account for both ligand flexibility and adaptive rearrangement of the renin binding site. The crystal structure of renin (PDB ID: 2FS4) was preprocessed using the Protein Preparation Wizard, where hydrogens were added, protonation states were optimized under the OPLS3 force field, crystallographic waters were removed, and the model was minimized with peripheral atom restraints. The ligand, N-CDAH, was subjected to energy minimization (MacroModel) and prepared with LigPrep prior to docking. The resulting poses were ranked using Glide XP, and the IFD score was calculated as GlideScore + 0.05 × Prime energy. The most favorable pose produced an IFD score of −11.149 kcal/mol, indicating a strong and specific binding affinity [9].

Interaction analysis demonstrated that the ligand is stabilized within the active site by a combination of hydrophobic enclosure, salt bridges, and hydrogen bonds. A dense network of hydrophobic residues, including Val31, Trp40, Leu76, Tyr78, Val83, Val106, Met109, Val122, Phe119, Ala117, Leu116, Phe114, Pro113, Met296, and Ala222, encased the cyclooctanoyl- moiety, generating van der Waals contacts that drive ligand encapsulation. Electrostatic stabilization was mediated by the formation of salt bridges between the protonated imidazole residue and the carboxylate groups of Asp33 and Asp219. In addition, the ligand engaged in directional hydrogen bonding: a direct hydrogen bond from the imidazole NH to Asp219, a second from the amide NH to Asp33, and two water-mediated hydrogen bonds involving Ala222 and Ser223.

This combination of hydrophobic packing, strong ionic interactions with the catalytic aspartate derivatives, and water-bridged hydrogen bonds produced an exceptionally favorable docking score, consistent with structural principles reported for high-affinity renin inhibitors [9,21]. In particular, dual salt bridge formation with Asp33 and Asp219 represents a hallmark of potent renin inhibition, while hydrophobic superpocket occupancy further enhances binding affinity.

**Figure 3:**
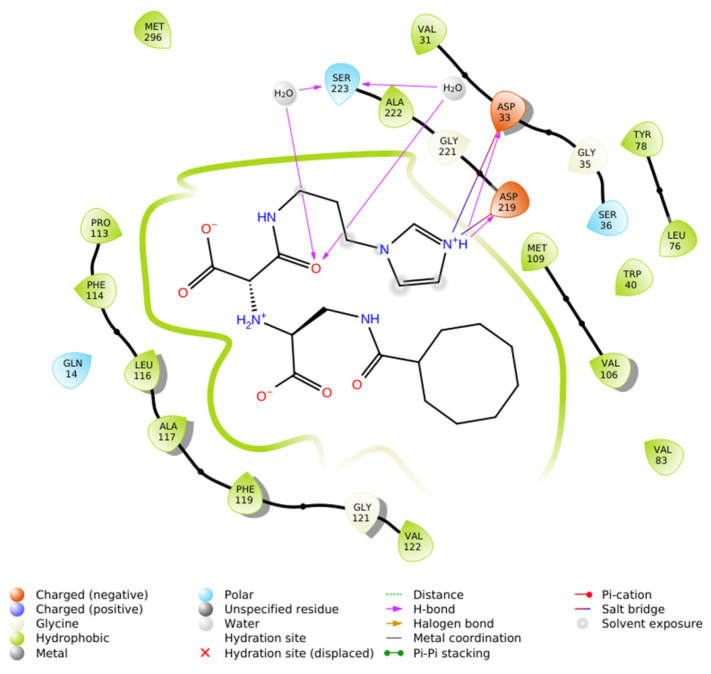
2D ligand interaction map of N-CDAH in the active site of renin (PDB ID: 2FS4), highlighting key non covalent contacts: hydrophobic enclosure by Val31, Trp40, Leu76, Tyr78, Val83, Val106, Met109, Val122, Phe119, Ala117, Leu116, Phe114, Pro113, Met296, and Ala222 (green); salt bridges between the ligand’s imidazolium center and Asp33/Asp219 (blue); water-mediated hydrogen bonds to Ser223 and Ala222 (purple arrows); and direct NH→O hydrogen bonds to Asp219 and Asp33.

In summary, the IFD results highlight that N-CDAH achieves extensive noncovalent stabilization within renin’s active site, combining hydrophobic, electrostatic, and hydrogen-bonding interactions in a manner consistent with rational renin inhibitor design. The details of these residue-level interactions are provided in Table 2, and the 2D interaction map is illustrated in Figure 3 [9,21].

#### Molecular Docking Analysis of Aliskiren with Renin (PDB ID: 2FS4). Induced-Fit Docking (IFD) Methodology in Maestro

**Figure 4:**
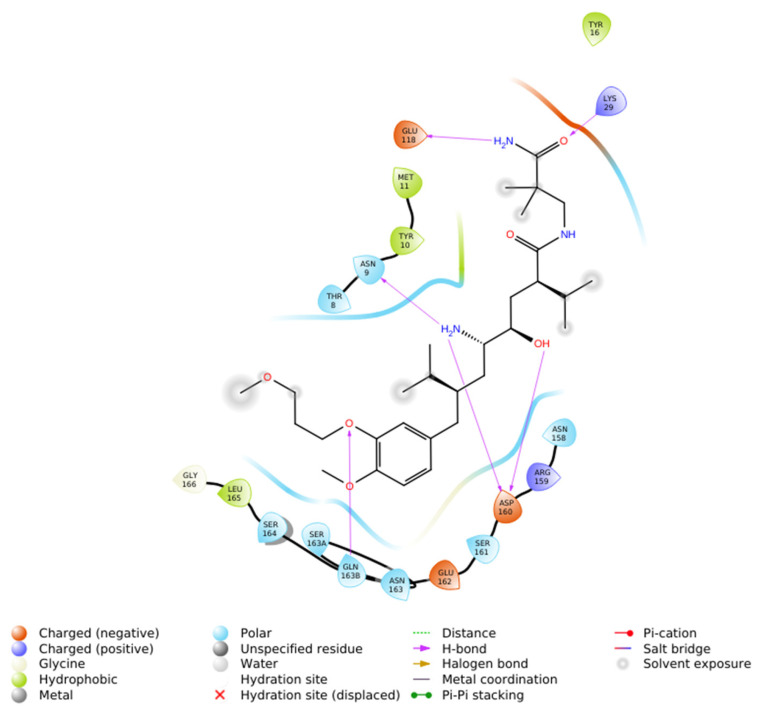
2D ligand interaction diagram of Aliskiren docked into renin (PDB ID: 2FS4) using Induced Fit Docking in Maestro—Schrödinger. The diagram illustrates hydrogen bonds with Asn9, Asp160, and Gln163B (purple arrows), polar contacts with Thr8, Asn9, Asn158, Ser161, Asp160, Ser164, and Gln163B (blue), hydrophobic contacts with Tyr10, Met11, Tyr18 and Leu165 (green) and proximity to negatively charged residues Glu118, Asp160, and Glu162 (brown).

The Induced Fit Docking (IFD) of Aliskiren with renin (PDB ID: 2FS4) was carried out using Maestro-Schrödinger, resulting in a docking score of −5.416 kcal/mol (Figure 4). Analysis of the binding pose revealed that Aliskiren establishes hydrogen bonds with Asn9, Lys29, Asp160, and Gln163B, which contribute significantly to the stabilization of the complex. Several residues were identified as polar contacts, including Thr8, Asn9, Asn158, Ser161, Asn163, Gln163B, and Ser164, indicating proximity interactions that may assist in orienting the ligand within the active site. In addition, hydrophobic interactions were observed with Tyr10, Met11, Tyr18 and Leu165, providing further stabilization through non-polar contacts. The docking also highlighted the presence of negatively charged residues, specifically Glu118, Asp160, and Glu162, in the vicinity of the ligand, which may contribute to electrostatic complementarity.

### 2.4. Comparative Binding of N-CDAH Versus Aliskiren to Renin (PDB ID: 2FS4)

Although Aliskiren has been withdrawn from the pharmaceutical market due to safety concerns, its extensively characterized binding mode continues to serve as a valuable reference for computational and structural analyses of renin inhibition. Both Aliskiren and N-CDAH demonstrate nanomolar-range affinity for renin, yet they achieve this through distinct interaction patterns and energetic contributions [5,9,22].

Collectively, these findings suggest that the binding of Aliskiren to renin (IF docking score −5.416 kcal/mol) is mediated through a cooperative network of hydrogen bonding, polar, hydrophobic, and electrostatic interactions, consistent with its role as a potent direct renin inhibitor but not as good as N-CDAH, which exhibited a substantially higher binding affinity with a docking score of −11.149 kcal/mol. The interaction analysis revealed that this compound forms an extensive and robust network of non-covalent interactions within the renin active site.

Specifically, in N-CDAH, hydrophobic enclosure was observed with residues Val31, Trp40, Leu76, Tyr78, Val83, Val106, Met109, Val122, Phe119, Ala117, Leu116, Phe114, Pro113, Met296, and Ala222, providing a tightly packed non-polar environment that enhances ligand stabilization. Moreover, two strong salt bridges were identified between the ligand’s imidazolium center and the acidic residues Asp33 and Asp219, while additional water-mediated hydrogen bonds connected the ligand to Ser223 and Ala222, further reinforcing the interaction network. Direct NH→O hydrogen bonds to Asp219 and Asp33 were also observed, contributing to enhanced binding specificity. Overall, N-CDAH–renin complex demonstrated the highest stability and most extensive range of stabilizing interactions, indicating superior inhibitory potential compared to withdrawn drug Aliskiren (Table 2).

### 2.5. Molecular Dynamics Simulations

#### 2.5.1. Molecular Dynamics Simulation of N-CDAH with Renin (PDB ID: 2FS4)

To further evaluate the dynamic stability and interaction profile of the renin–ligand complex, a 200 ns molecular dynamics (MD) simulation was carried out using the OPLS-2005 force field in Desmond (Maestro Schrödinger 2017-1). The ligand, N-CDAH, was embedded in an orthorhombic grid box of 20 Å, solvated with explicit TIP3P water molecules, and simulated under periodic boundary conditions to approximate physiological conditions. The system was neutralized with counter ions and equilibrated under the NPT ensemble at 310 K and 1.01 bar. A total of 200 trajectory frames were collected for structural and interaction analyses.

**Figure 5:**
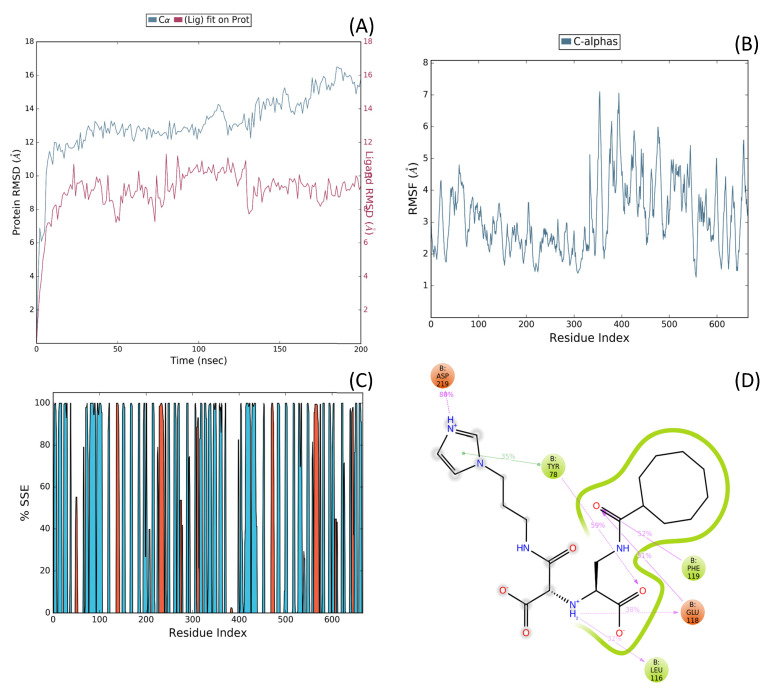
(**A**) Backbone and ligand RMSD versus time for the renin–N-CDAH complex, (**B**) Root mean square fluctuation (RMSF) profile of protein Cα atoms, highlighting residue-specific flexibility during the trajectory, (**C**) Protein–ligand interaction histogram for N-CDAH bound to renin during the 200 ns MD simulation (NPT ensemble, TIP3P solvent, 20 Å grid box). The color scale represents the secondary structure elements (SSE) throughout the simulation: blue corresponds to coil, red to α-helix, and cyan to β-sheet conformations. (**D**) 2D protein–ligand interaction diagram showing persistent hydrogen bonds and hydrophobic contacts of N-CDAH with Glu118, Phe119, Tyr78, and Asp219. In panel (**D**), green arrows indicate hydrogen bonds, purple dashed lines represent hydrophobic interactions, and the colored spheres highlight the interacting residues of renin (Glu118, Phe119, Tyr78, and Asp219). The ligand (N-CDAH) is shown in stick representation.

The simulation revealed that the renin–ligand complex remained structurally stable over the entire 200 ns trajectory. RMSD analysis of the protein Cα backbone plateaued at ~2.0–2.2 Å after 20 ns, confirming equilibration, while ligand RMSD remained consistently below 2.5 Å, indicating robust occupancy of the active site pocket (Figure 5A). RMSF profiling further demonstrated low per-residue fluctuations (<1.5 Å) for key active-site residues, including Val106, Met109, Phe114, Leu116, and Val122 (Figure 5B).

Protein–ligand contact histograms and 2D interaction diagrams (Figure 5C,D) revealed that the binding mode identified during docking was preserved throughout the simulation. Persistent hydrophobic contacts with Phe119 and Tyr78, coupled with stable salt bridges to Asp33 (~88% occupancy) and Asp219 (~91%), served as dominant stabilizing forces. In addition, water-mediated hydrogen bonds with Ser223 and Ala222 were detected in ~65% of frames, while direct hydrogen bonds with Asp33 and Asp219 contributed further to interaction stability, while Glu118 formed occasional transient contacts. These results emphasize a dual stabilization mechanism involving sustained hydrophobic enclosure and ionic anchoring, consistent with the highly negative IFD score.

A comparative perspective against Aliskiren highlights mechanistic differences. While N-CDAH relies heavily on hydrophobic encapsulation of its cyclooctanoyl- ring and hydrogen bonds, Aliskiren secures binding primarily through an extensive hydrogen-bonding network, with experimentally validated occupancies exceeding Met11, Asn9, Ser161, Gln280, Ala275, Val278. The quantitative differences between the two ligands are summarized in Table 3.

Taken together, these MD results reinforce that N-CDAH exhibits a stable and energetically favorable binding mode, complementary to but mechanistically distinct from Aliskiren. By maintaining structural stability through a synergistic combination of hydrophobic enclosure, salt bridge persistence, and water-mediated hydrogen bonds, the investigational compound (N-CDAH) demonstrates promising potential as a next-generation renin inhibitor.

To comprehensively assess the conformational stability and binding integrity of the renin–N-CDAH complex, molecular dynamics analysis extended beyond backbone Cα-RMSD evaluation. Residue-level RMSF calculations were performed to monitor local flexibility, with particular focus on side-chain mobility within the catalytic pocket. Time-resolved hydrogen-bond and salt-bridge occupancies, together with ligand RMSD profiles, were also analyzed to verify the persistence and geometric consistency of key binding interactions throughout the simulation. These parameters collectively confirmed that the backbone stability observed during the 200 ns trajectory was accompanied by low side-chain fluctuations at the active site and sustained intermolecular contacts, supporting the robustness of the docked binding mode under dynamic physiological conditions.

In addition to these stability metrics, the stochastic nature of molecular dynamics sampling was further addressed through time-resolved contact analysis. Using the Simulation Interaction Diagram and Event Analysis tools in Maestro (Schrödinger), we examined the probability distribution and dwell-time of key hydrogen-bond, salt-bridge, and hydrophobic interactions throughout the 200 ns trajectory. This analysis confirmed that the dominant contacts, including the salt bridges with Asp33 and Asp219 and hydrophobic interactions within the binding pocket, were not sporadic events but displayed high occupancy values and extended continuous residence periods, indicating sustained and statistically meaningful interaction persistence rather than transient frame-dependent fluctuations.

#### 2.5.2. Molecular Dynamics of Aliskiren with Renin (PDB ID: 2FS4)

**Figure 6:**
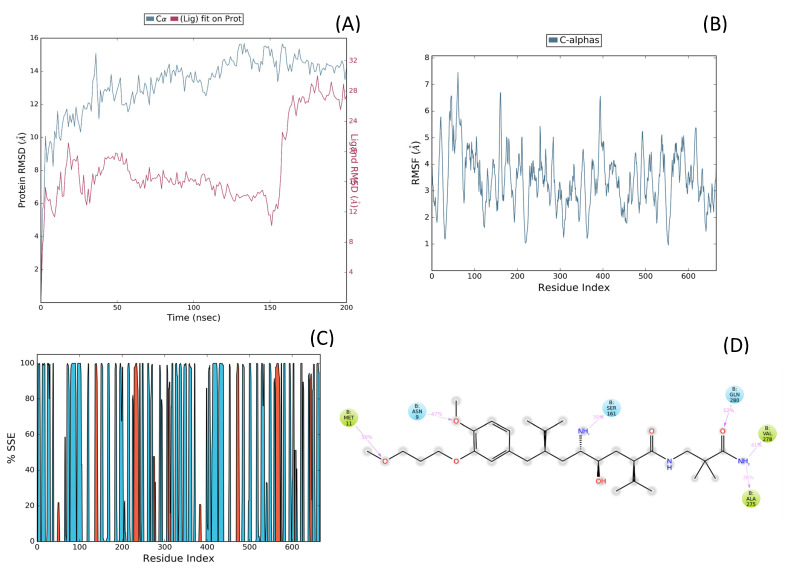
(**A**) Root Mean Square Deviation (RMSD) of the protein backbone (Cα atoms, blue) and ligand (red) during the 200 ns molecular dynamics simulation of the Aliskiren–Renin complex. The protein RMSD indicates structural stability after initial equilibration, while the ligand RMSD reflects its conformational adjustments within the binding pocket, (**B**) Root Mean Square Fluctuation (RMSF) plot of the Cα atoms of Renin throughout the 200 ns simulation. Peaks correspond to regions of higher flexibility, indicating loops and solvent-exposed areas, while stable regions correspond to secondary structure elements involved in ligand binding, (**C**) Secondary structure elements (SSE) percentage along the Renin residues during the simulation. The graph shows the persistence of α-helices (red) and β-sheets (cyan), demonstrating the overall structural conservation of the protein during the simulation. Τhe colored vertical bars correspond to different secondary structure elements (SSE): red indicates α-helices, cyan denotes β-sheets, and blue represents coil/loop regions along the Renin sequence during the simulation and (**D**) 2D interaction diagram summarizing the key residues of Renin involved in hydrogen bonding and hydrophobic contacts with Aliskiren during the MD simulation. green arrows represent hydrogen bonds between the ligand and renin, purple dashed lines denote hydrophobic interactions, and the colored spheres indicate the interacting amino acid residues involved in ligand stabilization. The interactions with residues Met11, Asn9, Ser161, Gln280, Val278, and Ala275 indicate stable binding and consistent contact frequencies throughout the trajectory.

Molecular dynamics (MD) simulations were performed to assess the structural stability and dynamic behavior of the Aliskiren–Renin complex (PDB ID: 2FS4). The system was solvated using the TIP3P water model within an orthorhombic grid box extending 20 Å from the complex, and simulations were conducted under NPT ensemble conditions at 310 K and 1.01 bar for 200 ns, generating 200 frames for analysis. As shown in Figure 6A, the RMSD of the protein Cα atoms stabilized around 14 Å after the initial equilibration phase, while the ligand RMSD fluctuated between 6 and 12 Å, indicating moderate conformational adaptation within the active site. The RMSF analysis (Figure 6B) revealed localized flexibility mainly at loop regions, with the binding pocket residues exhibiting minimal fluctuations, suggesting structural rigidity favorable for ligand retention. The secondary structure element (SSE) distribution (Figure 6C) confirmed the persistence of the protein’s native α-helices and β-sheets throughout the simulation, demonstrating overall structural stability. The 2D interaction profile (Figure 6D) identified key interactions between Aliskiren and residues Met11, Asn9, Ser161, Gln280, Val278, and Ala275, with contact frequencies ranging from 36% to 52%, indicating strong and persistent binding within the catalytic site of Renin.

Beyond the global backbone stability assessment obtained through Cα-RMSD, the MD analysis incorporated residue-level RMSF, side-chain mobility profiling, ligand RMSD, and hydrogen-bond/salt-bridge occupancy metrics to extract a more detailed picture of the dynamic binding landscape. These descriptors were used to evaluate whether key catalytic residues remained structurally confined, to monitor temporal stability of critical electrostatic and hydrophobic interactions, and to verify that the ligand maintained consistent positioning within the active site under simulation conditions. This integrated analysis ensured that the interpretation of binding stability was informed not only by backbone alignment but also by residue-specific flexibility and persistent interaction patterns throughout the 200 ns trajectory.

In parallel, the Aliskiren–Renin complex was analyzed under the same MD protocol to ensure a direct comparison of binding dynamics. RMSD and RMSF profiles indicated that the protein backbone remained structurally consistent over the 200 ns trajectory, while the ligand exhibited moderate fluctuations within the catalytic pocket, reflecting a more flexible binding mode relative to N-CDAH. To account for stochastic sampling behavior, probability distributions and dwell-time patterns for intermolecular contacts were evaluated using the Simulation Interaction Diagram and Event Analysis tools in Maestro. Although Aliskiren maintained persistent interactions with residues such as Met11, Asn9, Ser161, Gln280 and Val278, these contacts demonstrated shorter uninterrupted residence periods and more frequent disruption events compared to the dual salt-bridge and hydrophobic anchoring observed for N-CDAH. Overall, the dynamic profile supports a stable but more transient interaction pattern for Aliskiren, consistent with a less conformationally constrained fit within the renin binding cavity.

Overall, comparative molecular dynamics (MD) simulations highlighted distinct differences in the conformational stability and interaction persistence of the two renin–ligand systems. The N-CDAH –Renin complex exhibited a consistently compact and equilibrated structure over the entire 200 ns simulation, with the combined protein–ligand RMSD stabilizing around 16 Å, indicative of limited conformational drift and sustained binding geometry. In contrast, the Aliskiren–Renin complex displayed higher conformational flexibility, reaching an overall RMSD of approximately 32 Å, reflecting greater mobility of both the protein backbone and ligand scaffold within the binding cavity. The restrained fluctuations observed for the cyclooctanoyl- derivative suggest that its molecular framework effectively complements the topology and electrostatic environment of the catalytic pocket, maintaining optimal packing and minimizing solvent-induced destabilization. Furthermore, the RMSF profiles supported these observations, with the novel ligand inducing lower residue-level fluctuations, particularly at key catalytic positions (Asp33, Asp219, and Glu118), confirming a tighter structural confinement of the active site.

Beyond global stability, qualitative and quantitative interaction analyses reinforced the superior dynamic behavior of N-CDAH compared to Aliskiren. N-CDAH retained a robust dual stabilization mechanism, characterized by persistent hydrophobic encapsulation of its cyclooctanoyl- ring through contact with Phe119, Tyr78, and Val106, combined with long-lived salt bridges to Asp33 and Asp219 maintained in over 85–90% of simulation frames. In contrast, Aliskiren relied primarily on transient hydrogen bonds with residues such as Met11, Asn9, and Ser161, which exhibited lower contact persistence (36–52%). The integration of hydrophobic enclosure, electrostatic anchoring, and water-mediated hydrogen bonding in the novel ligand conferred a balanced and energetically favorable stabilization pattern that outperformed Aliskiren under identical NPT conditions. Collectively, these findings confirm that N-CDAH forms a more stable, compact, and dynamically resilient renin complex, underscoring its potential as a next-generation renin inhibitor with superior molecular stability and binding endurance relative to Aliskiren (Table 3).

### 2.6. Toxicity Predictions

The toxicological profile of N-CDAH (Table 4) was assessed using pkCSM and ProTox-II web-based predictive models, both of which provide widely validated in silico frameworks for early drug safety evaluation. Simulation of the Ames mutagenicity assay classified the compound as non-mutagenic, indicating minimal genotoxic risk. Importantly, no inhibition of hERG I or II potassium channels was detected, suggesting a low likelihood of cardiotoxic events, a common liability in small-molecule therapeutics.

The predicted oral LD_50_ in mice was approximately 302 mg/kg, corresponding to Toxicity Class IV (harmful if swallowed) under the Globally Harmonized System (GHS) classification. A Lowest Observed Adverse Effect Level (LOAEL) of 128 mg/kg bw/day was also estimated, consistent with moderate chronic exposure toxicity. Skin sensitization potential was absent, whereas a hepatotoxicity signal was flagged, indicating possible adverse effects on hepatic function upon repeated dosing.

Collectively, these findings suggest that N-CDAH exhibits a favorable preliminary safety profile, with minimal risks of mutagenicity, cardiotoxicity, and dermal sensitization. However, the prediction of hepatotoxic liability underscores the importance of integrating toxicity screening early in the computational design pipeline and the need for targeted experimental validation in cellular and animal models before clinical translation.

These computational toxicity predictions, when considered alongside the favorable binding stability observed in docking and molecular dynamics simulations, provide a balanced perspective on the therapeutic potential of N-CDAH, highlighting both its promise as a renin inhibitor and the necessity for further experimental validation of its safety profile.

### 2.7. Evaluation of an Important N-CDAH Diastereomer

**Figure 7:**
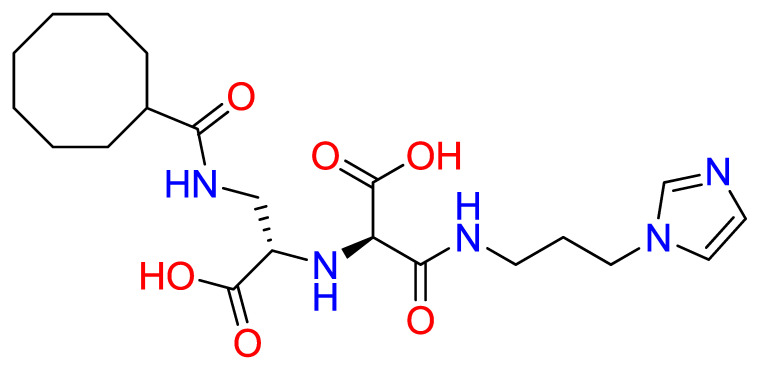
Chemical structure of N-CDAH diastereomer (blue: nitrogen atoms and their hydrogens, red: oxygen atoms and their hydrogens).

**Figure 8:**
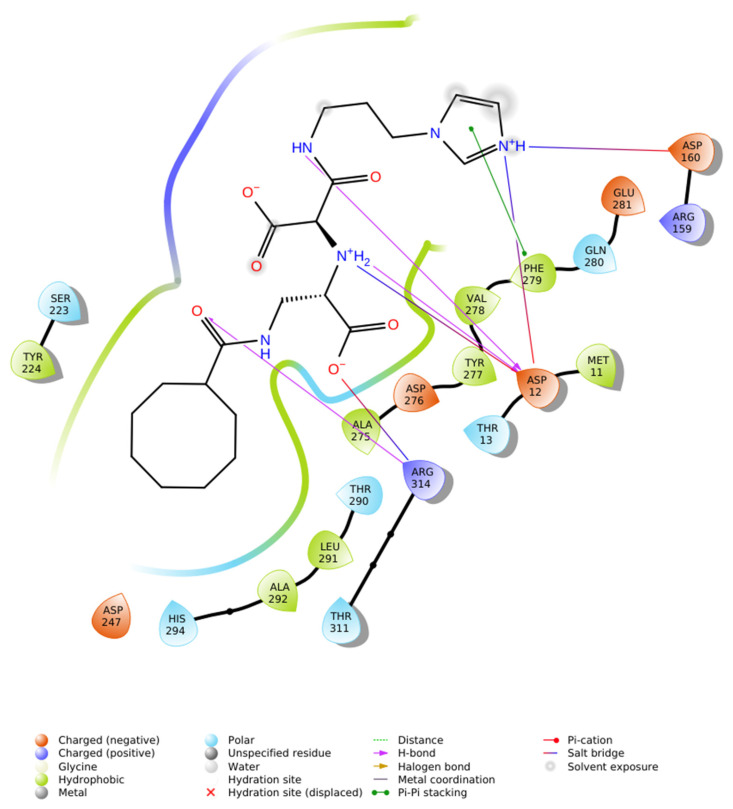
2D ligand interaction diagram of the N-CDAH diastereomer bound to renin (PDB: 2FS4). The diastereomeric configured ligand engages in hydrogen-bond interactions with Asp12, Asp160, and Arg314, while occupying a hydrophobic sub-pocket formed by Tyr224, Ala292, Leu291, Ala275, Tyr277, Val278, Phe279, and Met11. The ligand additionally forms hydrophobic bond with Phe279 and is surrounded by negatively charged residues (Asp247, Asp276, Asp12, Glu281, Asp160) and positively charged Arg159 and Arg314, consistent with electrostatic complementarity. Polar contacts are evident with His294, Thr311, Thr290, Thr13, and Ser223. Unlike the diastereomer, does not optimally align for dual salt-bridge anchoring with the catalytic Asp dyad, resulting in altered interaction geometry and reduced docking affinity (−7.443 kcal·mol^−1^).

To evaluate the stereochemical dependence of renin recognition, the diastereomeric analog of N-CDAH (Figure 7) was docked under identical conditions. The diastereomer retained the ability to occupy the catalytic pocket; however, its optimal pose exhibited a distinct interaction geometry compared to the native S-form. Specifically, the diastereomeric analog established hydrogen bonds with Asp12, Asp160, and Arg314, while forming hydrophobic contacts with Tyr224, Ala292, Leu291, Ala275, Tyr277, Val278, Phe279, and Met11, including a hydrophobic bond with Phe279. Electrostatic complementarity was maintained through proximity to negatively charged residues (Asp247, Asp276, Asp12, Glu281, Asp160) and positively charged Arg159 and Arg314, and additional polar contacts were observed with His294, Thr311, Thr290, Thr13, and Ser223 (Figure 8).

Despite these stabilizing contacts, the reversed stereochemistry altered the spatial projection of the di-acidic motif, preventing optimal formation of the dual salt-bridge anchoring with the catalytic Asp dyad that characterizes the diastereomer. This disruption of cooperative ionic binding resulted in a less favorable docking affinity (−7.443 kcal·mol^−1^) and a sub-optimal fit within the active-site cleft. These findings confirm that renin recognition is strongly chirality-dependent and support the rational selection of the -S-configured scaffold for inhibitor design.

To complement the structural analysis of the diastereomeric analog of N-CDAH, its full ADME–toxicity profile was evaluated using SwissADME and pkCSM. The diastereomer preserved the same fundamental physicochemical characteristics as the parent S-form (MW 451.52 g/mol; 5 H-bond donors; 8 acceptors; 14 rotatable bonds; TPSA 162.65 Å^2^) and exhibited a nearly identical lipophilicity distribution across all logP models (consensus logP = −0.44). SwissADME classified the compound as highly water-soluble (ESOL logS = −1.10; class: very soluble), with low GI absorption, no BBB permeation, and P-gp substrate behavior—mirroring the profile of N-CDAH. No inhibition was predicted for any CYP isoform, and the bioavailability score remained 0.11, indicating no stereochemical impact on metabolic liability.

The pkCSM predictions further confirmed that the diastereomer displays a largely comparable pharmacokinetic pattern. Despite a lower predicted water solubility (logS = −4.856), intestinal absorption was moderately high (72.4%), and vdss was low (logVDss = −0.317), reflecting limited tissue distribution. The compound was predicted not to inhibit P-gp or any CYP isoform and to act as a CYP3A4 substrate, consistent with the parent scaffold. Clearance was moderate (log CL = 2.05), while toxicity endpoints showed no mutagenicity (Ames = No), absence of hERG inhibition, and lack of skin sensitization. Similarly to N-CDAH, hepatotoxicity was the only flagged liability. LD50 (3.084 mol/kg) and LOAEL predictions were also aligned with the S-form. Altogether, these results demonstrate that stereochemical inversion does not significantly alter the ADME or toxicity profile of the scaffold, reinforcing that the diminished binding affinity of the diastereomer arises from suboptimal geometric complementarity within the renin active site rather than intrinsic pharmacokinetic drawbacks.

### 2.8. Evaluation of Structurally Related Analogs of N-CDAH

**Figure 9:**
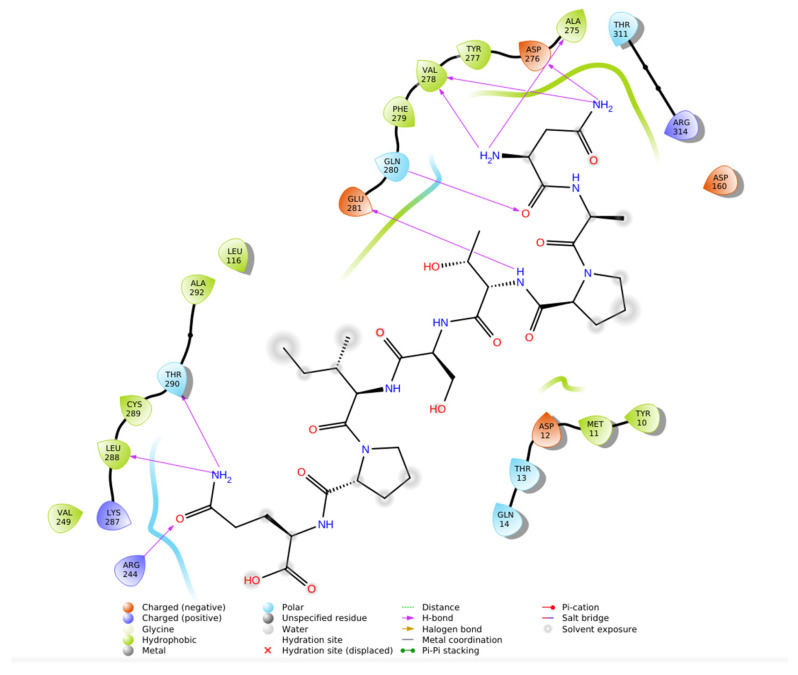
2D ligand interaction diagram of Davunetide derivative bound to renin (PDB: 2FS4). Davunetide derivative forms a hydrogen-bond network with Ala275, Asp276, Val278, Gln280, Glu281, Leu288, and Thr290, stabilizing the compound backbone along the catalytic groove. The ligand is embedded in an extensive hydrophobic environment comprising Val249, Tyr277, Val278, Phe279, Ala275, Ala292, Leu291, Leu288, Met11, and Tyr10. Electrostatic complementarity is evident from nearby negatively charged residues (Asp12, Asp160, Asp276, Glu281) and positively charged residues (Arg246, Lys287, Arg314). Despite lacking dual catalytic Asp anchoring as in N-CDAH, Davunetide derivative shows productive accommodation within the S1/S3 pocket, consistent with its strong predicted affinity (−8.760 kcal·mol^−1^).

**Figure 10:**
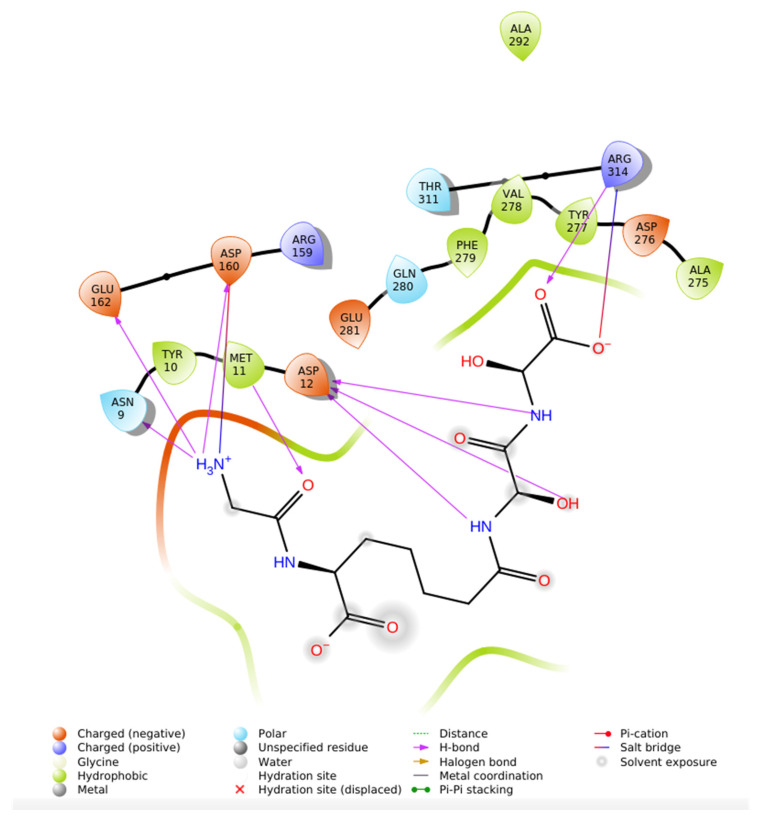
2D ligand interaction diagram of the (S)-2-(2-aminoacetamido)-7-(((R)-2-(((R)-carboxy(hydroxy)methyl)amino)-1-hydroxy-2-oxoethyl)amino)-7-oxoheptanoic acid analog docked to renin (PDB: 2FS4). The ligand forms hydrogen bonds with Asn9, Asp12, Asp160, Glu162, and Arg314, supporting stable positioning within the catalytic cleft. Hydrophobic contacts are observed with Tyr10, Met11, Phe279, Val278, Tyr277, Ala275, and Ala292, reinforcing anchoring along the S1/S3 pocket. The ligand is surrounded by negatively charged residues (Glu162, Asp160, Asp12, Glu281, Asp276) and positively charged residues (Arg159, Arg314), reflecting strong electrostatic complementarity. Additional polar interactions with Asn9, Gln280, and Thr311 further stabilize the binding pose. The interaction pattern is consistent with its docking affinity (−8.808 kcal·mol^−1^).

To further validate the structural rationale of the N-CDAH scaffold and explore the influence of related compound motifs on renin binding, a ligand-based similarity search was performed using the SwissSimilarity platform. Three top-ranking analogs displaying high 2D/3D chemical similarity to N-CDAH were selected for comparative docking: Davunetide derivative, (S)-2-(2-aminoacetamido)-7-(((R)-2-(((R)-carboxy(hydroxy)methyl)amino)-1-hydroxy-2-oxoethyl)amino)-7-oxoheptanoic acid, and Perindoprilat. All compounds were docked to renin (PDB: 2FS4) using identical computational settings to the main ligand to ensure a consistent comparative framework. As shown in Figure 9, Davunetide derivative established multiple stabilizing hydrogen bonds and extensive hydrophobic interactions within the S1/S3 region, resulting in a strong docking affinity (−8.760 kcal·mol^−1^). The second analog demonstrated comparable or slightly improved affinity (−8.808 kcal·mol^−1^), forming a dense network of hydrogen bonds with Asn9, Asp12, Asp160, Glu162, and Arg314, as illustrated in Figure 10, and maintaining effective hydrophobic and electrostatic complementarity within the catalytic groove. A comparative overview of the chemical structures of the selected analogs alongside their corresponding Maestro Schrödinger 2021 docking scores is presented in Table 5, providing a concise visualization of their relative binding performance.

**Figure 11:**
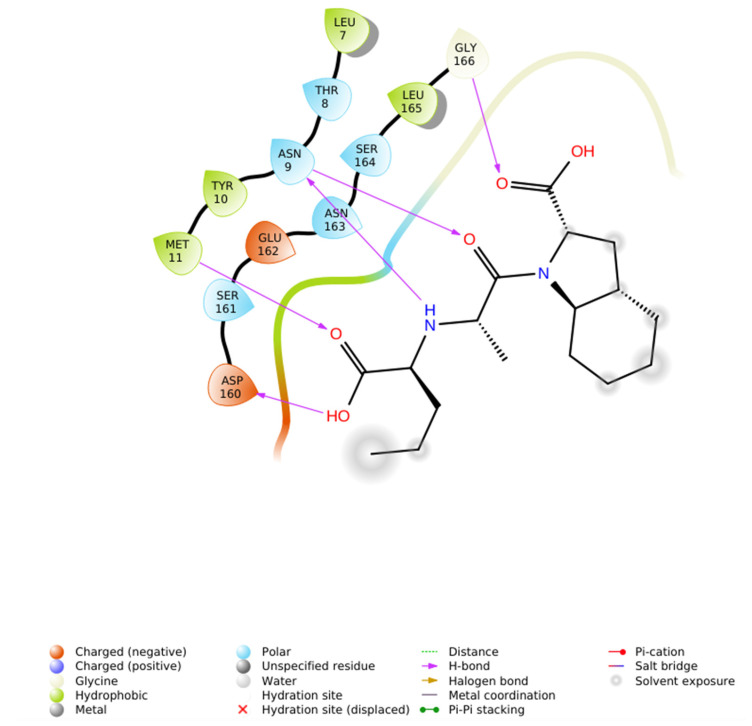
2D ligand interaction diagram of Perindoprilat that interacts with renin (PDB through H-bonds (Asn9, Met11, Gly166), hydrophobic contacts (Leu165, Met11, Tyr10, Leu7), polar residues (Ser161, Asn163, Ser164, Asn9, Thr8), and limited negative-charge contacts (Asp160, Glu162), consistent with weak affinity (−5.412 kcal/mol).

In contrast, Perindoprilatdemonstrated substantially weaker binding to renin (−5.412 kcal·mol^−1^), with its interaction pattern limited to a small number of shallow hydrogen bonds and hydrophobic contacts near the pocket entrance. The 2D ligand interaction diagram (Figure 11) reflects this reduced complementarity, showing absence of deep insertion into the catalytic dyad environment and a lack of strong anchoring interactions with key acidic residues such as Asp33/Asp219. Collectively, these analog comparisons confirm that the N-CDAH scaffold occupies an optimal structural space for renin inhibition, characterized by a balanced combination of hydrogen bonding, acidic moieties, and hydrophobic enclosure. The results reinforce the design strategy and demonstrate that compound analogs with appropriate steric and electrostatic properties can achieve high affinity, whereas scaffolds optimized for ACE inhibition, such as Perindoprilat, fail to engage the active-site architecture required for potent renin binding.

## 3. Discussion

The integration of induced fit docking modeling, molecular dynamics, and computational toxicology in this study underscores the power of in silico strategies to guide early-stage protein–ligand evaluation in drug discovery. By systematically comparing N-CDAH with the benchmark inhibitor Aliskiren, we highlight how modern computational approaches can not only rationalize binding affinity and stability but also anticipate pharmacokinetic and safety liabilities that contributed to Aliskiren’s market withdrawal. This multi-tiered computational workflow provides molecular-level insights into renin inhibition, enabling the identification of novel scaffolds with optimized hydrophobic interactions, salt bridge formation, and persistent hydrogen bonding within the S1/S3 pocket of renin. Furthermore, by combining drug design principles with toxicity predictions, our study illustrates how computational methods can bridge the gap between structure-based drug design and translational pharmacology, offering a cost-effective route to de-risk and prioritize candidates for experimental validation.

### 3.1. Molecular Docking as a Structural Probe

Two complementary docking strategies—AutoDock and Schrödinger-Induced Fit Docking—consistently placed the lipophilic cyclooctanoyl- moiety into the hydrophobic S1/S3 superpocket of renin, establishing π-π stacking with Tyr78, Phe114, and Phe119. The agreement across independent docking engines strengthens confidence in the predicted binding pose. Binding affinities of −8.08 kcal/mol (AutoDock) and −11.149 kcal/mol (IFD) are much better than Aliskiren’s calculated free energy (−5.416 kcal/mol), suggesting that this scaffold may approach nanomolar inhibition. Importantly, the occupation of aromatic superpockets through hydrophobic enclosure aligns with established design strategies for protease inhibitors, where lipophilic substituents are engineered to maximize van der Waals stabilization.

Notably, the induced-fit docking score of N-CDAH (−11.149 kcal/mol) compared to Aliskiren (−5.418 kcal/mol) represents a dramatic improvement—exceeding 5 kcal/mol—and constitutes the central computational finding of this study. Such a pronounced energy gap underscores the superior geometric complementarity and optimized interaction network of the designed scaffold within the renin active site, validating the rationale behind its structure-based engineering.

### 3.2. Pharmacokinetic Constraints and Drug-likeness

ADME predictions reveal that the ligand is highly polar and water-soluble (log S ≈ −1 to −2.9) with favorable safety liabilities (no CYP inhibition, no Ames mutagenicity). This metabolic stability represents a key advantage gained through the computational design approach, directly addressing a major drawback of Aliskiren’s extensive CYP3A4-mediated metabolism. Yet, analogous to Aliskiren, its large TPSA (>160 Å^2^), high hydrogen-bond donor/acceptor counts, and P-gp substrate status predict low gastrointestinal absorption and poor oral bioavailability. These findings highlight a recurring challenge in peptidomimetic drug design: balancing target-directed polarity with physicochemical constraints on permeability. Computational pharmacokinetic profiling therefore informs future rational design interventions such as acyl chain modifications, scaffold rigidification, or prodrug strategies to improve bioavailability without sacrificing binding affinity.

From a physicochemical perspective, a key design success lies in the lipophilicity balance between the two inhibitors. Whereas Aliskiren is distinctly hydrophobic (LogP ≈ +4.15), N-CDAH exhibits near-neutral lipophilicity (Consensus LogP = −0.44; pkCSM = 0.36). This substantial reduction in hydrophobicity demonstrates the intentional tuning of polarity to improve solubility and metabolic compatibility while maintaining potent renin binding. Such a hydrophilicity-driven design strategy effectively differentiates N-CDAH from its withdrawn predecessor.

### 3.3. Dynamic Stability of N-CDAH in Complex with Renin

The 200 ns MD simulations reinforced the docking results by demonstrating a stable renin-ligand complex throughout the trajectory. RMSD stabilization (~2.0 Å backbone) and consistent ligand positioning support binding robustness. Contact analysis revealed a dual stabilization mode: sustained hydrophobic enclosure (Phe119, Tyr78, Val106) and ionic/hydrogen bond persistence (Asp219, Glu118, Ser223). This balance between nonpolar packing and polar complementarity is consistent with renin’s catalytic architecture and mirrors principles underlying the design of next-generation transition-state analog inhibitors. The ability of MD to reveal temporal persistence of specific salt bridges and solvent-mediated H-bonds provides deeper mechanistic insight beyond static docking poses, showcasing how MD complements protein design by validating binding hypotheses under near-physiological conditions.

When compared to the Aliskiren–Renin (PDB ID: 2FS4) complex, which exhibited significantly higher conformational deviations with a combined protein–ligand RMSD reaching approximately 32 Å, the N-CDAH complex demonstrated notably improved dynamic stability, with an RMSD of only about 16 Å. This substantial reduction in structural deviation indicates tighter conformational restraint and enhanced interaction persistence within the active site. Consequently, the novel cyclooctanoyl- derivative displays a superior dynamic profile, maintaining its binding integrity throughout the 200 ns simulation and suggesting a more favorable stability pattern than Aliskiren under comparable NPT simulation conditions.

### 3.4. Toxicity Forecasting and Translational Implications

In silico toxicology (pkCSM, ProTox-II) predicted a non-mutagenic, non-cardiotoxic profile, with a moderate oral LD_50_ (~302 mg/kg, GHS Class IV). However, potential hepatotoxicity was flagged, underlining the need for experimental hepatocyte assays or in vivo validation. This finding is noteworthy in the context of Aliskiren’s withdrawal, as off-target effects and metabolic burdens can derail otherwise potent inhibitors. By integrating toxicity screening early in the computational pipeline, protein design efforts can be de-risked before resource-intensive synthesis and experimental campaigns are initiated.

### 3.5. Diastereomeric Effects on Renin Binding

The diastereomeric comparison provided additional insight into the stereochemical determinants that govern productive renin inhibition. Despite retaining several hydrogen bonds and hydrophobic contacts within the catalytic cleft, the diastereomeric analog of N-CDAH was unable to reproduce the precise geometric alignment required for optimal interaction with key residues of the Asp33/Asp219 catalytic dyad. The chains of the chiral center altered the projection of the di-acidic groups, resulting in suboptimal orientation and weaker anchoring within the S1/S3 subsites. Consequently, the diastereomer exhibited reduced docking affinity and an overall less stable interaction profile compared with N-CDAH. These findings highlight the stringent stereochemical requirements of renin’s active site and confirm that N-CDAH represents the stereochemically privileged orientation, providing the appropriate three-dimensional architecture for strong, persistent binding.

### 3.6. Comparative Analysis of Structurally Related Analogs

Evaluation of compound analogs identified through SwissSimilarity [23] further clarified the structural attributes necessary for high-affinity renin engagement. Both Davunetide derivative and the (S)-2-(2-aminoacetamido)-7-(((R)-2-(((R)-carboxy(hydroxy)methyl)amino)-1-hydroxy-2-oxoethyl)amino)-7-oxoheptanoic acid analog demonstrated docking scores comparable to or slightly surpassing N-CDAH, supported by extensive hydrogen-bond networks and favorable hydrophobic complementarity within the S1/S3 region. However, neither analog achieved the balanced interaction profile or the deep, dual anchoring within the catalytic dyad that characterize N-CDAH. In contrast, Perindoprilat showed substantially weaker binding, forming only superficial interactions consistent with its optimization toward ACE rather than renin. Collectively, these comparisons demonstrate that while related compound scaffolds can partially recapitulate key interaction motifs, none fully reproduce the synergistic combination of ionic anchoring, hydrophobic enclosure, and steric complementarity exhibited by N-CDAH, reinforcing its designation as the most structurally and energetically optimized inhibitor candidate among the evaluated analogs.

### 3.7. Translational Perspective

Taken together, the results highlight N-CDAH as a computationally validated renin inhibitor scaffold, offering a differentiated pharmacological profile compared to Aliskiren. Its aromatic pocket-filling and polar interaction balance align with structure-based design principles, while its physicochemical challenges call for formulation or medicinal chemistry innovation. Importantly, this study illustrates how computational protein design approaches—from flexible docking to long-timescale MD—can guide the prioritization of scaffolds prior to costly experimental campaigns.

The findings herein position N-CDAH as a computationally validated renin inhibitor scaffold, offering a differentiated pharmacological profile compared to Aliskiren. From a translational perspective, N-CDAH could serve as a blueprint for re-engineering renin inhibitors that retain high enzymatic potency while exhibiting improved pharmacological safety. Its predicted non-CYP metabolism implies compatibility with commonly prescribed RAAS-modulating agents such as ACE inhibitors and angiotensin-receptor blockers, potentially enabling safe combination therapies that eluded Aliskiren. Moreover, the modular compound architecture facilitates straightforward chemical diversification—through acyl-chain length variation, backbone cyclization, or imidazole substitutions—allowing fine-tuning of absorption and distribution without compromising target affinity. If future in vitro and in vivo studies corroborate these computational insights, N-CDAH or its analogs may emerge as clinically viable renin inhibitors with an enhanced therapeutic index.

Together, the markedly superior IFD binding energy and the deliberate reduction in lipophilicity define the dual design triumphs of this study—computationally stronger binding with improved physicochemical harmony.

### 3.8. Limitations and Future Directions

Despite the promising findings, several limitations must be acknowledged. Docking and MD-derived binding free energies provide relative rather than absolute accuracy; rigorous free energy perturbation (FEP+) [24] or thermodynamic integration could refine affinity estimates. Water-mediated interactions, while captured qualitatively, require entropic decomposition for full energetic characterization. Pharmacokinetic predictions remain model-based and necessitate experimental ADME assays for validation. Finally, the hepatotoxicity flag emphasizes the importance of preclinical toxicology studies.

Future computational studies should incorporate enhanced sampling techniques (e.g., metadynamics, accelerated MD [25]) to probe conformational adaptability of the renin active site, as well as QM/MM methods to elucidate protonation dynamics. From a design perspective, systematic SAR-guided optimization—including variation in acyl chain length, heteroaromatic substitutions, and backbone constraints—may be necessary to improve bioavailability while maintaining potency. Ultimately, integration of these computational refinements with enzyme inhibition assays, crystallographic validation, and preclinical safety models will be essential to advance this scaffold toward clinical translation.

This study further underscores the transformative role of computational protein design in antihypertensive drug discovery. By leveraging in silico experimentation, it is now possible to screen, refine, and prioritize candidate scaffolds long before chemical synthesis, dramatically reducing attrition rates and development costs. The concordance between docking, MD, and ADME/tox outputs for N-CDAH validates the predictive reliability of this integrated approach. The structural determinants identified here—hydrophobic superpocket occupancy, dual salt-bridge formation, and solvent-mediated hydrogen-bond persistence—provide actionable design principles for future optimization cycles aimed at improving permeability and oral exposure.

## 4. Materials and Methods

Aliskiren was included as a reference inhibitor and processed using the same computational workflow applied to N-CDAH. Molecular docking was performed with AutoDock Vina and Schrödinger Maestro, while molecular dynamics simulations were carried out in Desmond through the Maestro interface under identical system preparation and simulation settings. This ensured full methodological consistency for all comparative analyses.

### 4.1. Ligand Preparation

N-CDAH was constructed in ACD/ChemSketch 12.0.2. [26] and subjected to energy minimization using the MMFF94 force field. The ligand geometry and conformational space were optimized to ensure accurate representation of its binding-competent state. Physicochemical and ADME properties—solubility, permeability, lipophilicity, and metabolic stability—were predicted via the SwissADME [9,12], and pkCSM [13,15], web servers. These in silico tools enabled early assessment of drug-likeness and toxicity liabilities, critical for drug design.

Toxicity predictions were also performed using the ProTox-II web server [27], a machine-learning–based platform integrating over 50,000 experimentally annotated toxicological data points. The tool provides multi-endpoint toxicity classification, including LD_50_ estimation, hepatotoxicity, cytotoxicity, immunotoxicity, carcinogenicity, and mutagenicity (Ames test), using deep learning models and structural similarity fingerprints. Both N-CDAH and Aliskiren were submitted in SMILES format, and toxicity outputs were generated under default settings. The resulting predictions were used to compare acute toxicity, chronic exposure (LOAEL), organ-specific toxicities, and overall toxicity class according to the Globally Harmonized System (GHS).

Structural analogs of N-CDAH were identified using the SwissSimilarity web platform [23]. The query molecule was submitted in SMILES format, and similarity searching was performed using FP2 and ECFP4 fingerprints to capture both substructure and extended circular pharmacophore similarity. Candidate analogs were ranked according to their global similarity score, and the top-scoring molecules were selected for further processing. All retrieved compounds were downloaded in 2D format, converted to 3D using LigPrep (OPLS3e force field, through Maestro Schrodinger 2021), and subsequently subjected to the same docking and interaction-analysis workflow applied to N-CDAH, enabling direct structural and energetic comparison.

### 4.2. Target Protein Preparation

The crystal structure of human renin (PDB ID: 2FS4, resolution 2.2 Å) was retrieved from the Protein Data Bank. All crystallographic water molecules and non-protein heteroatoms were removed. For AutoDock Vina 1.5.7., polar hydrogens were added, Kollman charges assigned using AutoDockTools 1.5.7. (MGLTools) [16], and the receptor saved in PDBQT format. For Schrödinger docking, the Protein Preparation Wizard added hydrogens, assigned protonation states at pH 7.4, optimized hydrogen bonding networks, and minimized the structure under OPLS3 force field restraints, constraining peripheral atoms. This ensured biologically relevant active-site protonation and accurate electrostatic mapping for renin’s catalytic dyad.

### 4.3. Molecular Docking Protocols

AutoDock Vina 1.5.7.: A grid box encompassing the catalytic Asp219, Asp295, and His287 residues was defined. Ten poses were generated, ranking by binding affinity, via AutoDockTools; the top pose was selected for interaction analysis in PyMOL 2.6. A blind docking screening was subsequently performed in AutoDock Vina, covering the full protein surface. The highest-scoring poses consistently localized near Asp33/Asp219, validating the targeted grid docking strategy used in the workflow.

Schrödinger Induced Fit Docking (IFD): Docking was performed for the poses selected after energy minimization of the ligand (Minimization, Macromodel) and further preparation of it using LigPrep in Maestro Schrödinger 2021. The top 20 poses underwent Glide docking and glide redocking with Glide XP to yield IFD scores (GlideScore + 0.05 × Prime energy) [28]. The combined rigid-receptor and induced-fit approaches allowed evaluation of both optimal ligand orientations and protein conformational adaptability, capturing the dynamic plasticity of the renin binding site.

### 4.4. Comparative Analysis

Docking affinities, key ligand–protein interactions, and predicted ADME profiles for the novel cyclooctanoyl- derivative were benchmarked against Aliskiren’s post withdrawal data. Pharmacokinetic parameters and docking reference values for Aliskiren were taken from DrugBank, SwissADME, and literature sources to ensure a consistent and transparent comparison framework. This comparative methodology enabled systematic identification of structural and pharmacological features potentially responsible for improved safety and efficacy profiles of the designed derivative.

### 4.5. Molecular Dynamics Simulation Protocols

Molecular dynamics (MD) simulations were performed using the Desmond module of the Schrödinger suite to assess the dynamic stability of the renin–ligand complexes. The OPLS-2005 force field was applied for all atoms. Each protein–ligand complex was embedded in an orthorhombic TIP3P explicit solvent box with a 10 Å buffer, and counterions were added to neutralize the system. The solvated complexes were subjected to energy minimization and stepwise equilibration under NPT ensembles. Production simulations were run for 200 ns at 300 K and 1 atm, employing the Nose–Hoover thermostat and Martyna–Tobias–Klein barostat (Schrödinger, LLC, New York, NY, USA). Trajectories were analyzed for root mean square deviation (RMSD), root mean square fluctuation (RMSF), radius of gyration (Rg), hydrogen-bond occupancy, and protein–ligand contact persistence [29]. This protocol allowed detailed evaluation of conformational flexibility and long-term stability of the designed cyclooctanoyl- derivative compared with Aliskiren within the renin active site.

**Figure 12:**
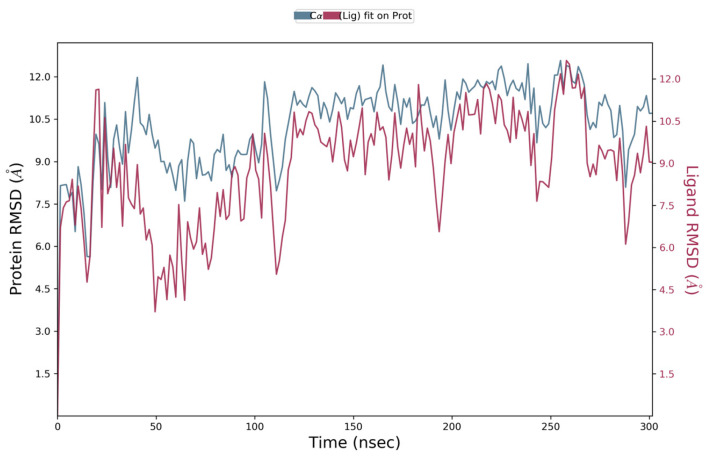
Protein (Cα, blue) and ligand (red) RMSD profiles for the renin–N-CDAH complex during a 300 ns molecular dynamics simulation (NPT ensemble, TIP3P solvent, 310 K). Both trajectories reach equilibrium after approximately 20–30 ns and remain stable thereafter, confirming that the conformational stability observed at 200 ns is representative of the equilibrated state of the system.

To further validate the conformational stability of the renin–ligand complex and justify the choice of simulation timescale, an additional 300 ns molecular dynamics (MD) simulation was conducted using the Desmond module (Maestro Schrödinger 2021) under identical NPT conditions (310 K, 1.01 bar, TIP3P solvent). The root mean square deviation (RMSD) trajectories for both protein backbone (Cα) and ligand heavy atoms were monitored across the extended simulation window. The RMSD values plateaued after approximately 20–30 ns and remained stable throughout the 300 ns run, indicating that the conformational ensemble achieved equilibrium well within the initial 200 ns (Figure 12). This result supports the adequacy of the 200 ns trajectory length used for all comparative analyses.

## 5. Conclusions

The present work proposes (S)-3-((3-(1H-imidazol-1-yl)propyl)amino)-2-(((S)-1-carboxy-2-(cyclooctanecarboxamido)ethyl)amino)-3-oxopropanoic acid (N-CDAH) as a rationally designed and computationally supported candidate with the potential to improve upon the withdrawn direct renin inhibitor Aliskiren. Through an integrated in silico workflow combining molecular docking, induced-fit modeling, molecular dynamics simulations, and predictive ADME/toxicity profiling, this study provides convergent structural and energetic evidence that the new lipophilic cyclooctanoyl- derivative may interact with renin more efficiently and stably than its predecessor.

Across independent docking platforms, N-CDAH consistently achieved stronger predicted affinity for renin (AutoDock Vina = −8.08 kcal·mol^−1^; Maestro IFD = −11.149 kcal·mol^−1^) compared to Aliskiren (−5.418 kcal·mol^−1^). The notably improved scoring profile reflects enhanced non-covalent interactions and superior geometric complementarity between the flexible compound scaffold and the catalytic pocket. Dual salt-bridge formation with Asp33 and Asp219, together with extensive hydrophobic enclosure by Tyr78, Phe114, Phe119, Val106, and Met109, suggests a dual stabilization mechanism—electrostatic anchoring combined with hydrophobic sealing—potentially supporting increased specificity and residence time. In contrast, Aliskiren primarily relies on a more transient hydrogen-bond network and lacks persistent dual ionic engagement, which may contribute to its lower predicted binding strength and reduced dynamic persistence.

The 200 ns molecular dynamics trajectories further support this binding hypothesis. The renin–N-CDAH complex maintained a compact, equilibrated structure (protein RMSD ≈ 2 Å; ligand RMSD < 2.5 Å), with low active-site RMSF values and sustained contacts throughout the simulation, including persistent salt bridges (>85% occupancy), water-mediated hydrogen bonds, and π–π stacking with Tyr78 and Phe119. Conversely, the renin–Aliskiren complex exhibited greater structural drift (protein RMSD ≈ 14 Å; ligand RMSD 6–12 Å) and less stable interaction persistence (36–52%), consistent with comparatively reduced confinement within the catalytic pocket. These observations provide a strong computational rationale suggesting that N-CDAH may exhibit enhanced dynamic and thermodynamic stability relative to Aliskiren.

In silico pharmacokinetic and toxicity predictions further indicate a potentially improved safety profile for N-CDAH. The compound is smaller (451.5 Da vs. 551.8 Da), more hydrophilic (LogP ≈ 0 vs. +4.1), and demonstrates greater predicted solubility (logS ≈ −1 to −2.9). Importantly, N-CDAH is not predicted to inhibit major cytochrome P450 isoforms, addressing a key limitation associated with Aliskiren’s drug interaction risk. Although predicted low oral bioavailability remains a challenge, its solubility and metabolic stability profile provide a rational foundation for future formulation strategies or prodrug development. Predictive toxicity models classify N-CDAH as moderately safe (LD50 ≈ 302 mg·kg^−1^, GHS Class IV) with no mutagenicity or hERG cardiotoxicity signals, though potential hepatotoxicity warrants follow-up experimental evaluation.

Taken together, these multi-tier computational findings suggest that N-CDAH could represent a next-generation renin inhibitor scaffold capable of addressing limitations that contributed to Aliskiren’s withdrawal. Its balanced physicochemical properties, dual stabilization binding mode, and benign predicted metabolic profile collectively support its candidacy as a promising renin inhibitor. While the conclusions herein are predictive and must be validated experimentally, the consistency across orthogonal computational analyses provides strong justification for further biochemical, structural, and pharmacological evaluation.

Furthermore, evaluation of the diastereomeric analog and structurally similar compounds obtained through SwissSimilarity confirmed that none of the additional molecules reproduced the combined energetic, structural, and dynamic advantages observed for N-CDAH. This comparative screening indicates that the designed compound outperforms both its diastereomeric counterpart and structurally related analogs, reinforcing its identification as the most promising scaffold within the explored chemical space.

In summary, this work illustrates how rational molecular design supported by integrated computational screening may identify candidates with improved binding behavior and safety potential relative to existing scaffolds. N-CDAH emerges from these analyses as a promising renin-inhibitor scaffold that merits experimental confirmation and optimization in the pursuit of next-generation RAAS-targeted therapeutics.

## Figures and Tables

**Table 1:** Comparative pharmacokinetic and toxicity parameters of N-CDAH, its diastereomer, and Aliskiren derived from SwissADME and pkCSM predictions. The two stereochemical variants of N-CDAH exhibit nearly identical physicochemical and ADME profiles, indicating minimal stereochemical influence on intrinsic pharmacokinetic behavior. In contrast, Aliskiren displays markedly higher lipophilicity, very poor aqueous solubility, and CYP3A4-related interaction liabilities. Overall, N-CDAH demonstrates the most favorable balance of solubility, metabolic safety, and absence of CYP inhibition among the three evaluated structures.

Property	N-CDAH (SwissADME)	N-CDAH (pkCSM)	Diastereomer (SwissADME)	Diastereomer (pkCSM)	Aliskiren (SwissADME)	Aliskiren (pkCSM)
Molecular weight (Da)	451.52	451.52	451.52	451.52	551.77	551.77
Consensus LogP	−0.44	0.3621	−0.44	-	~4.1	2.59
HBD/HBA	5/8	5/7	5/8	-	6/9	6/9
Rotatable bonds	14	12	14	-	14	12
TPSA (Å^2^)	162.65	-	162.65	-	~174.7	-
Water solubility	Very soluble (logS = −1.10)	logS = −2.892	Very soluble (logS = −1.10)	logS = –4.856	Very low (logS ≈ −6 to −7)	logS ≈ −7
GI absorption	Low	18.7%	Low	72.4%	Low	17.4%
BBB permeation	No	logBB = −1.276	No	logBB = −1.283	No	logBB = −2.03
P-gp substrate	Yes	Yes	Yes	Yes	Yes	Yes
CYP inhibition	None	None	None	None	CYP3A4 issues	CYP3A4 substrate
Total clearance	-	-	-	2.05	-	-
Ames mutagenicity	-	No	-	No	-	No
Hepatotoxicity	-	Yes	-	Yes	-	Yes (reported clinically)
hERG inhibition	-	No	-	No	-	Possible risk (literature)
LD50 (oral, rat)	-	-	-	3.084 mol/kg	-	-
LOAEL	-	-	-	1.03	-	-
Bioavailability score	0.11	-	0.11	-	~0.17	-

**Table 2:** Unified comparative interaction table for N-CDAH and Aliskiren bound to renin (PDB: 2FS4), integrating results from AutoDock Tools and Maestro Schrödinger 2021. AutoDock reveals the fundamental aromatic and hydrophobic anchors of N-CDAH, while Maestro identifies a more complete interaction network, including water-mediated hydrogen bonds and dual salt bridges with Asp33 and Asp219. Aliskiren exhibits a weaker pattern dominated by transient hydrogen bonds and minimal hydrophobic packing. Collectively, the unified table highlights the superior and more cohesive interaction profile of N-CDAH relative to the clinical inhibitor.

Residue	AutoDock Tools—N-CDAH	Maestro 2021—N-CDAH	Maestro 2021—Aliskiren
Tyr78	H, π-π	H	-
Phe119	π-π	H	-
Phe114	π-π	H	-
Val106	H	H	-
Ala117	H	H	-
Pro113	H	H	-
Pro42	H	-	-
His56	P	-	-
Val31	-	H	-
Trp40	-	H	-
Leu76	-	H	-
Val83	-	H	-
Met109	-	H	-
Val122	-	H	-
Ala222	-	H + HB(w)	-
Ser223	-	HB(w)	-
Asp33	-	HB + I	-
Asp219	-	HB + I	-
Thr8	-	-	P
Asn9	-	-	HB + P
Tyr10	-	-	H
Met11	-	-	H
Tyr18	-	-	H
Asp160	-	-	HB + P
Asn158	-	-	P
Ser161	-	-	P
Gln163B	-	-	HB + P
Ser164	-	-	P
Leu165	-	-	H
Glu118	-	-	-
Glu162	-	-	-

Legend: H = Hydrophobic, HB = Hydrogen bond, HB(w) = water-mediated hydrogen bond, I = Ionic (salt bridge), P = Polar, π-π = aromatic stacking.

**Table 3:** Key N-CDAH and Aliskiren–Renin interactions from molecular dynamics.

Parameter	N-CDAH	Aliskiren
Simulation time (ns)	200	200
Water model	TIP3P	TIP3P
Box type/size (Å)	Orthorhombic/20 Å	Orthorhombic/20 Å
Ensemble/Conditions	NPT (310 K, 1.01 bar)	NPT (310 K, 1.01 bar)
Total frames analyzed	200	200
Protein RMSD (Cα)	~8.5 Å (stabilized)	~14 Å (stable after equilibration)
Ligand RMSD	~6.2 Å	6–12 Å (moderate conformational drift)
Average RMSF (Cα)	2.8 Å	3.2 Å
Most flexible regions	Surface loops	Loop regions near binding pocket
Secondary structure retention	>85% α/β maintained	>80% α/β maintained
Dominant interactions	Tyr78, Leu116, Glu118, Phe119, Asp219	Met11, Asn9, Ser161, Gln280, Val278, Ala275
Key hydrogen bond occupancy (%)	35–60%	36–52%
Overall complex stability	Stable with moderate ligand adaptation	Stable binding, strong persistence within catalytic pocket

**Table 4:** Comparative Toxicity Profile of N-CDAH and Aliskiren (pkCSM, ProTox-II).

Toxicity Parameter	N-CDAH	Aliskiren
Ames test (mutagenicity)	Negative (non-mutagenic)	Negative (non-mutagenic)
LD_50_ (oral)	~302 mg/kg (mouse)—Class IV	~1000–2000 mg/kg (rat)—Class IV
Chronic LOAEL	~128 mg/kg/day	~0.5–1.0 log mg/kg/day
hERG I/II inhibition	No	No
Hepatotoxicity	Yes (predicted)	Yes (clinically reported)
Skin sensitization	No	No

**Table 5:** Chemical structures and docking scores of the three N-CDAH analogs identified via SwissSimilarity and evaluated through molecular docking against renin (PDB: 2FS4) using Maestro Schrödinger 2021. The table summarizes the structural features of each analog and their corresponding predicted binding affinities (blue: nitrogen atoms and their hydrogens, red: oxygen atoms and their hydrogens).

Compound	Chemical Structure	Docking Score (kcal/mol)
Davunetide Derivative	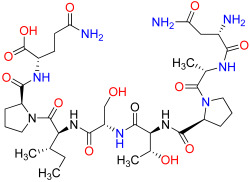	−8.760
(S)-2-(2-aminoacetamido)-7-(((R)-2-(((R)-carboxy(hydroxy)methyl)amino)-1-hydroxy-2-oxoethyl)amino)-7-oxoheptanoic acid	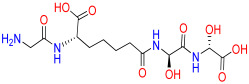	−8.808
Perindoprilat	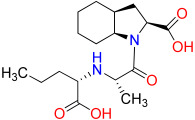	−5.412

## Data Availability

The original contributions presented in this study are included in the article. Further inquiries can be directed to the corresponding authors.
